# Magnetic field induced flow pattern reversal in a ferrofluidic Taylor-Couette system

**DOI:** 10.1038/srep18589

**Published:** 2015-12-21

**Authors:** Sebastian Altmeyer, Younghae Do, Ying-Cheng Lai

**Affiliations:** 1 Institute of Science and Technology Austria (IST Austria), 3400 Klosterneuburg, Austria; 2 Department of Mathematics, KNU-Center for Nonlinear Dynamics, Kyungpook National University, Daegu, 702-701, South Korea; 3 School of Electrical, Computer and Energy Engineering, Arizona State University, Tempe, Arizona, 85287, USA

## Abstract

We investigate the dynamics of ferrofluidic wavy vortex flows in the counter-rotating Taylor-Couette system, with a focus on wavy flows with a mixture of the dominant azimuthal modes. Without external magnetic field flows are stable and pro-grade with respect to the rotation of the inner cylinder. More complex behaviors can arise when an axial or a transverse magnetic field is applied. Depending on the direction and strength of the field, multi-stable wavy states and bifurcations can occur. We uncover the phenomenon of *flow pattern reversal* as the strength of the magnetic field is increased through a critical value. In between the regimes of pro-grade and retrograde flow rotations, standing waves with zero angular velocities can emerge. A striking finding is that, under a transverse magnetic field, a *second reversal* in the flow pattern direction can occur, where the flow pattern evolves into pro-grade rotation again from a retrograde state. Flow reversal is relevant to intriguing phenomena in nature such as geomagnetic reversal. Our results suggest that, in ferrofluids, flow pattern reversal can be induced by varying a magnetic field in a controlled manner, which can be realized in laboratory experiments with potential applications in the development of modern fluid devices.

Reversal of a fluid flow upon parameter changes or perturbation is closely related to intriguing phenomena such as geomagnetic reversal, a drastic change in a planet’s magnetic field where the positions of magnetic north and south are interchanged^1^. Typically, for a planet there is dynamo action in which convection of molten iron in the core produces electric currents, generating geomagnetic field. The reversal of the molten iron flow direction can cause the geomagnetic field to switch the poles. Computational fluid models incorporating the interaction between electromagnetism and fluid dynamics, e.g., in the Earth’s interior, were developed^2^,^3^,^4^,^5^,^6^ to account for the complete flip flop of the geomagnetic field which can occur within a few 10000 years of each other. There was also a recent experimental study of liquid metal in which global field reversals occurred at irregular time intervals^7^. To study the dynamical mechanism and controlled generation of flow reversal is of interest.

In this paper, we report magnetic-field induced flow pattern reversals in the classic Taylor-Couette system (TCS)^8^, which can exhibit a large number of flow structures of distinct topologies and has been an experimental and computational paradigm for investigating many fundamental phenomena in fluid dynamics for decades^9^,^10^,^11^,^12^,^13^. In our study, we consider ferrofluid^14^ in between the cylinders, the dynamics of which constitute an area of interest with a variety of applications ranging from embedded fluidic devices in computer hard drives to laboratory experiments designed to probe into the fundamentals of geophysical flows^15^,^16^. Generally, a ferrofluid consists of a conventional fluid with embedded nano-sized, magnetized particles. In the absence of any external magnetic field, the magnetic moments of the nanoparticles are randomly oriented, leading to zero net magnetization for the entire fluid. In this case, the magnetized nanoparticles have little effect on the physical properties of the fluid, e.g., its density and viscosity. However, an external magnetic field can have a drastic effect on the fluid and its dynamics. Usually, the axial component of the magnetic field does not tend to change the physical properties of the fluid but it can shift the various bifurcation points of the flow structures and patterns. For example, when the magnetic field is entirely axial, the onset of the basic rotational state in TCS tends to shift toward a larger value of the bifurcation parameter^17^,^18^. The transverse component of the external magnetic field, however, additional to a shift alters the physical properties of the fluid dramatically^17^,^19^,^20^, leading to characteristic or even fundamental changes in the underlying hydrodynamics. Especially, we demonstrate that, as the magnitude of the magnetic field is systematically changed, the system can exhibit repeated reversals in the ferrofluidic wavy vortex flow pattern in between the two rotating cylinders.

The TCS with conventional fluid or with ferrofluid but without external magnetic field typically possesses a large number of solutions with distinct dynamical properties, some of which can coexist in a wide range of parameters^11^. For example, it was shown earlier that different wavy states can occur^21^,^22^ for the same parameters (e.g., the Reynolds number 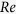 and the radius ratio between the two cylinders). It was also discovered that, as *Re* is increased, the wavy flows can become supercritical, and the wave speed and the angular velocity tend to decrease monotonically and approach an asymptotic value^23^,^24^,^25^. It was also discovered that for TCS with a wide gap between the inner and outer cylinders (e.g., radius ratio below 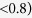, the waviness is usually dominated by low azimuthal modes of low speed and angular frequency. For TCS with a ferrofluid, as an external magnetic field is applied, one might intuitively expect the basic rotational state to be stabilized with an increasing angular velocity. However, our study demonstrates that the flow dynamics can become much more complicated than this intuitive picture would suggest. We note that, recent studies on the transition to turbulence in ferrofluidic flows^26^ suggest the possibility to control turbulence through an applied magnetic field.

Our work was motivated by the recent discovery in TCS with conventional fluid that the azimuthal waviness can change the direction of the rotation^27^. In particular, as the radius ratio is increased, the wavy flows can become co-rotating, and a reversal in the direction of rotation can occur, which is typically associated with a change in the azimuthal symmetry. For example, accompanying the flow pattern reversal, transitions from three-fold to two-fold or even to one-fold azimuthal waviness were observed. Curiosity thus demanded that we ask what might happen when the conventional fluid is replaced by a ferrofluid and a magnetic field is present. We find that, as the strength of the magnetic field is increased, flow pattern reversal can occur. However, in contrast to the TCS with conventional fluid, the reversals are in fact *smooth* transitions *without any change in the azimuthal symmetry of the underlying flows*. In particular, with variation in the strength of either axial or transverse magnetic field, the fluid motion can change from pro-grade to retrograde wavy flow patterns separated by stable interim, standing-wave solutions with zero angular velocities. These standing waves are part of an other class of flow states, mixed-ribbon^28^. For example, the azimuthal contributions to the wavy flows of azimuthal wave numbers 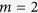 and 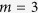 are maintained with variations only in their strength. A more striking phenomenon is that, as the strength of a transverse magnetic field is increased, the flow pattern can change its direction twice - from pro-grade to retrograde and back to pro-grade, a phenomenon that has not been observed in any study of the TCS. Our results suggest that flow pattern reversals in the ferrofluidic TCS can be controlled through an external magnetic field, which is not only fundamentally interesting but also relevant for practical development of novel fluid devices.

## Results

### Nomenclature

In this work we focus on toroidally closed wavy solutions, meaning that the axisymmetric Fourier mode, i.e. the azimuthal wavenumber, 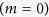 is always the strongest/largest. The corresponding flows possess different (pronounced) azimuthal modes for non-axisymmetric contributions (*m* ≠ 0). For most wavy flows studied, there are two different azimuthal modes, 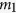 and 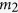, typically with unequal contributions. To specify the flow patterns, we use the following notations: 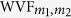 for a wavy vortex flow solution with dominant (major) azimuthal wavenumber 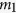 and subordinated (minor) azimuthal wavenumber 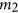 (apart from the strongest mode 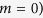. Of particular interest are wavy solutions WVF_2,3_, WVF_3,2_, and WVF_2_, with the last having azimuthal wavenumber 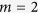 only in addition to the axisymmetric mode. It is worth mentioning that all calculated wavy flows are *stable*. However, for the parameter regimes considered the Taylor-vortex flow (TVF) solutions are unstable. The magnetic field strength can be characterized by the Niklas parameter (see **Methods**). In particular, for axial or transverse field, this parameter is 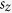 or 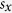, respectively, where 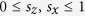 in the present work. The velocity and vorticity fields are 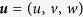 and 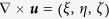, respectively.

### Wavy structures in absence of any magnetic field

**Figure 1:**
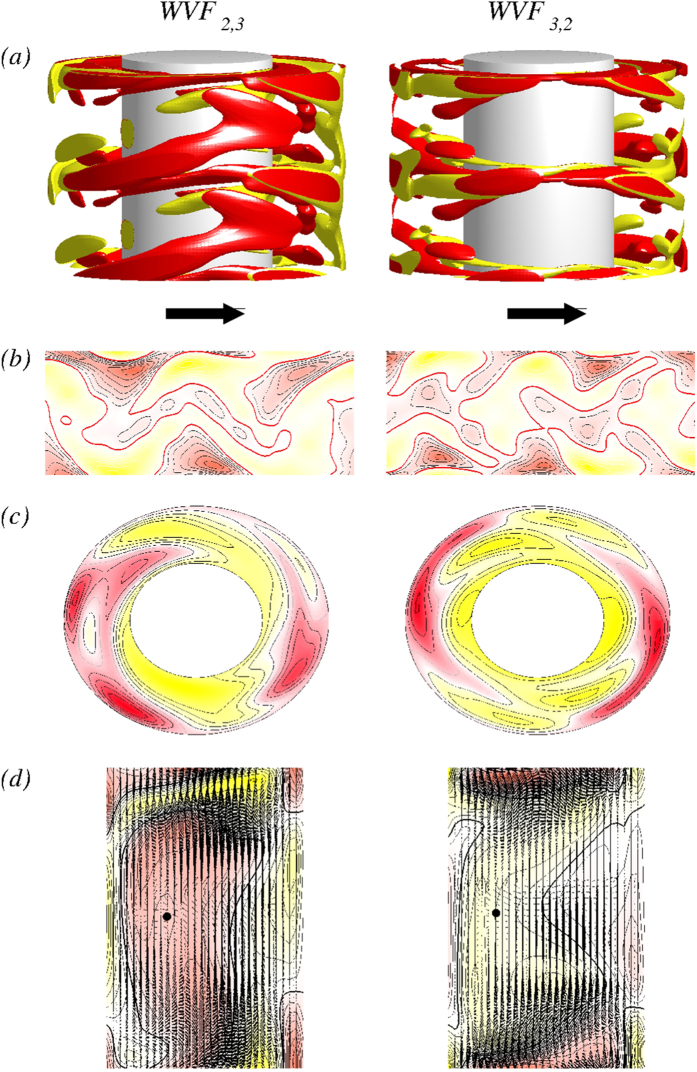
Flow structures in absence of any magnetic field. Flow states of WVF_2,3_ (left panels) and WVF_3,2_ (right panels) for 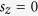 and 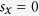. (***a***) Isosurfaces of 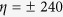. Red (yellow) color indicates positive (negative) vorticity. For clear visualization, two periods are plotted in the axial direction. The arrows below the snapshots illustrate the rotational direction (appearing laterally on the cylinders), which is pro-grade for both wavy flows. Note, here and in the following the rotation direction always refers to the pattern rotation. Contours of the flow structures: (***b***) radial velocity 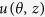 on an unrolled cylindrical surface in the annulus at mid-gap, (***c***) azimuthal vorticity 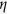 in the 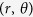 plane at mid-height, and (***d***) vector plots 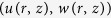 of the radial and axial velocity components in a constant *θ* = plane, including the azimuthal vorticity 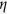. The two points mark the centerline of vortices within the illustrated plane. Note that this position is *θ*-dependent but the azimuthal averaged position for WVF_3,2_ is closer to the inner cylinder than for WVF_2,3_. All contours are color coded from red (dark gray - minimum) to yellow (light gray - maximum). See also movie files movie1.avi and movie2.avi in SMs.

We first briefly describe the flow structures and properties in absence of any magnetic field: *s_x_* = 0 and *s_z_* = 0. For the parameter regime considered, there are two characteristically distinct coexisting wavy states, which persist even in the presence of an magnetic field. Both wavy flows include the azimuthal modes *m* = 2 and *m* = 3, but the relative weights of the modes are different: for WVF_2,3_ the *m* = 2 mode dominates but for WVF_3,2_ the *m* = 3 mode dominates. Figure 1 illustrates both flow patterns: WVF_2,3_ in the left panels and WVF_3,2_ in the right panels. Figure 1(a) shows the isosurface plots of 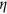 for two axial wavelengths, a three-dimensional representation of the flow with the interactions between the azimuthal modes *m* = 2 and *m* = 3. These contributions to the resulting waviness can be seen from the contour plots of the radial velocity 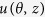, as shown in Fig. 1(b) on an unrolled cylindrical surface in the annulus at mid-gap. The more complex pattern of WVF_3,2_ illustrates a stronger influence of the *m* = 3 contribution than WVF_2,3_. Both flow patterns exhibit a pronounced *m* = 2 contribution, which is visible in the azimuthal vorticity 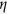 in the 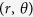 plane at mid-height, as shown in Fig. 1(c). The centerline of the vortices for WVF_3,2_ is located closer to the inner cylinder than the one for WVF_2,3_, which can also be seen in the vector plots  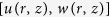 of the radial and axial velocity components in a constant *θ* plane, as shown in Fig. 1(d). In order to gain insights, we highlight the two points that mark the centerline of vortices within the illustrated plane. However, it is important to note that the position as well as the entire flow profile are *θ*-dependent. Nonetheless, the azimuthal averaged position (about the whole cylinder) of the centerline of vortices for WVF_3,2_ is closer to the inner cylinder than for WVF_2,3_. [See movie files movie1. avi and movie2.avi in Supplementary Materials (SMs), where the pro-grade rotation can be identified in 3D isosurfaces and contours of the respectively structure].

**Figure 2:**
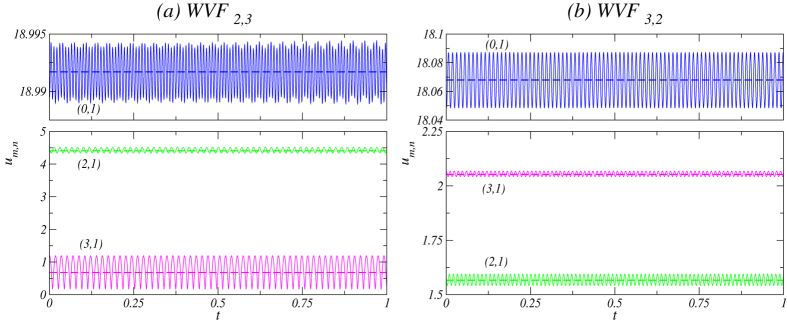
Time evolution of dominant flow amplitudes. Flow states of WVF_2,3_ (left panels) and WVF_3,2_ (right panels) for 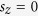 and 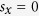. Shown are time-dependent [and time-averaged (dashed lines)], dominant amplitudes 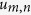 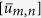 of the radial velocity field at mid-gap contributed by the axisymmetric mode 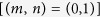, the 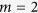 mode 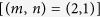, and the 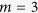 mode 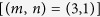, respectively.

With respect to the comparative values of *m* between the flow states WVF_2,3_ and WVF_3,2_, we show in Fig. 2 variations with time *t* of the dominant flow field mode amplitudes *u*_0,1_, *u*_2,1_, and *u*_3,1_, which are present in WVF_2,3_ [2(*a*)] and WVF_3,2_ [2(*b*)]. Since we focus on wavy flows with toroidal closed symmetry, the azimuthal component *m* = 0 is always dominant (cf., axis scaling in Fig. 2) and the wavy modulation actually originates from the higher azimuthal modes (e.g., *m* = 2 and *m* = 3. Depending on the amplitudes of these modes, especially the amplitude ratio, the combined solution can exhibit predominantly a 2-fold (WVF_2,3_) or a 3-fold (WVF_3,2_) symmetry. To characterize the corresponding states we used the time-averaged value *m*. For example, in Fig. 2(a), 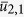 is significantly larger than 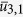 so that the flow is designated as WVF_2,3_. In general, in the TCS various combinations of different azimuthal wave numbers are possible, which can result in complex, mixed states such as mixed-cross-spiral patterns^28^. Differing from such mixed states, the predominant mode in our system is always the azimuthal symmetric 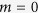 mode, so the underlying flow structures remain toroidally closed. It is possible that, when some parameters are systematically varied, a WVF_3,2_ solution can change to WVF_3,2_, and vice versa.

### Pro-grade and retrograde flows in presence of a magnetic field

In TCS the rotational direction of the flow pattern is defined in terms of the relative speed of rotation of the inner and outer cylinders (i.e., the Reynolds numbers 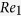 and 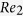, respectively). Without any magnetic field, the most common case is that the flow pattern follows the rotational direction of the inner cylinder. Only for quite strong counter-rotations (e.g., larger 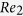 value as compared to the value of 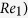 does the flow pattern follow the rotational direction of the outer cylinder. We fix both 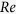 values with the ratio 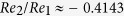. Without any magnetic field, both wavy flows are “normal” in the sense that they rotate along the rotational direction of the inner cylinder, i.e., pro-grade motion.

**Figure 3:**
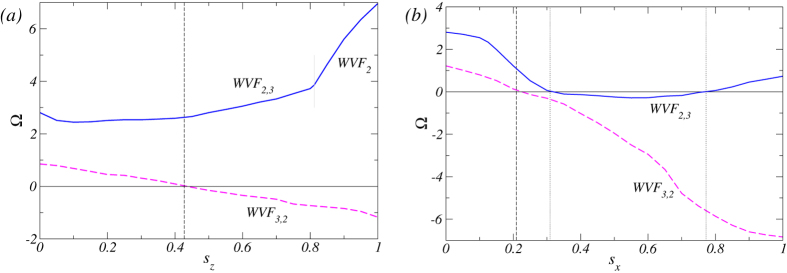
Pro-grade and retrograde wavy flows in presence of a magnetic field. Variations in the angular velocity Ω of the wavy states in the presence of axial [*s*_*z*_≠0 but 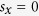, (***a***)] and transverse [*s*_*x*_≠0 but 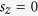, (***b***)] magnetic field. Vertical dotted and dashed lines indicate the points of non-rotating flow pattern, i.e., standing waves, which are the reversal points of the motion for WVF_2,3_ and WVF_3,2_, respectively. The horizontal black line indicates zero angular velocity. For axial field strength 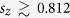, there is wavy flow WVF_2_ without any 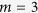 contribution, where the short thin gray line marks the bifurcation point. With axial field only WVF_3,2_ changes its propagating direction for 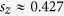. With transverse field both wavy flows change their propagation directions: WVF_3,2_ at 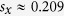 and WVF_2,3_ twice, first for 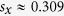 to retrograde and second for 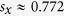 back to prograde behavior. For WVF_2,3_ the minimal angular velocity 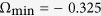 is achieved for 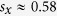.

Figure 3 illustrates the variations in the angular velocity Ω of the wavy pattern as the magnetic field strength 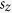 or 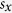 is increased. For an axial magnetic field, the WVF_2,3_ flow is pro-grade for all values of 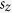 examined, as shown in Fig. 3(a). In the regime of low 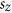 values, as 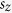 is further decreased the angular velocity Ω increases continuously with visibly steeper slope after the bifurcation point 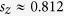, which results from the disappearance of the 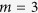 mode so that the flow corresponds to a pure WVF_2_ solution. [See movie file movie3. avi in SM.] This is essentially the same results obtained previously^27^, taking into consideration the fact that a magnetic field tends to stabilize the basic rotational state^17^,^20^. In the large 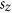 regime, the angular velocity of WVF_3,2_ decreases monotonously which, for 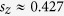, becomes zero, effectively turning WVF_3,2_ into a standing wave. Note, that these are special kind of standing waves, mixed-ribbon^28^. For 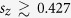, the flow pattern becomes retrograde, rotating in the opposite direction to that of the inner cylinder [See movie file movie4.avi in SM for WVF_3,2_ at 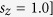. For a transverse magnetic field, WVF_3,2_ exhibits qualitatively similar changes with the transition from pro-grade to retrograde motions occurring at 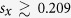, as shown in Fig. 3(b) [See movie file movie7.avi in SM for WVF_3,2_ at 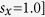. The difference between the cases of transverse and axial magnetic field is that, for the former, the magnitude of the angular velocity Ω for larger 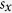 values is much higher than that for the latter.

For the flow WVF_2,3_, the behavior of the angular velocity Ω is dramatically different for different field directions. For a transverse field, as 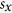 is increased, Ω decreases continuously, vanishes for 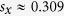, and becomes negative as 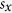 is increased further [See movie file movie5.avi in SM for WVF_2,3_ at 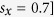, similar to the behavior of the flow WVF_3,2_. As 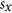 is further increased, Ω reaches minimum at 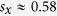 and begins to increase from the minimum afterwards. For 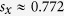, Ω is zero again and becomes positive as 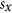 is increased [See movie file movie6.avi in SM for WVF_2,3_ at 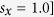. Thus the flow pattern of WVF_2,3_ reverses *twice*: from pro-grade to retrograde and back to pro-grade. This is quite different from the situation of an axial magnetic field, where no flow pattern reversal takes place for WVF_2,3_ and the pattern remains to be pro-grade. Another difference is that, in the presence of a transverse magnetic field, there is always contribution to the flow pattern from the 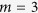 mode. (The relative contributions from the 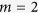 and 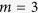 modes will be detailed below.) Increasing or decreasing the 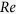 values, we expect the curves for Ω to move upwards or downwards, respectively. For certain value of *Re*, 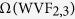 can no longer reach zero. In such as case, no flow pattern reversal would occur. For instance, in the parameter regime of weakly counter-rotating cylinders, we find that the curve for WVF_2,3_ Fig. 2(d) will move move away from Ω = 0 towards large, positive values of Ω, rendering prograde the underlying flow.

### Bifurcations with magnetic field

**Figure 4:**
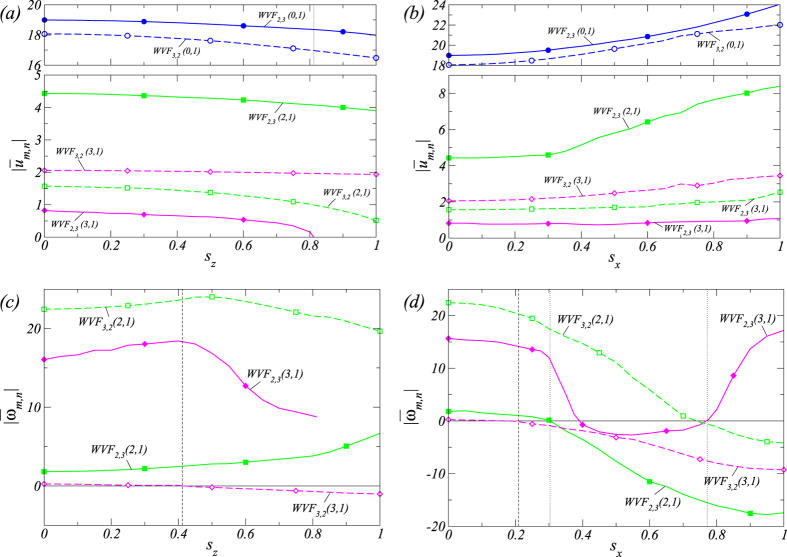
Bifurcation with the strength of magnetic field. Variation of WVF_2,3_ (WVF_2_) and WVF_3,2_ with axial (left column) and transverse (right column) magnetic field strength. Shown are (***a***,***b***) time-averaged, dominant amplitudes 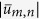 of the radial velocity field at mid-gap contributed from the axisymmetric mode 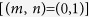, the 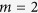 mode 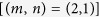, and the 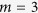 mode 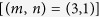, respectively. (***c***,***d***) The corresponding time-averaged frequency  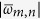 of the complex mode amplitudes 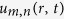. Symbols are for eye guidance (the same for subsequent figures), but calculations were typically done for many more parameter values. All presented solutions are stable. The vertical dashed (dotted) lines indicate the critical magnetic field strength in 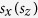 at which the WVF_3,2_ (WVF_3,2_) flow changes its direction of rotation (cf. Fig. 3): 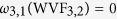 for 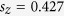, 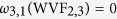 for 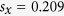, 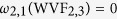 for 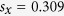, and 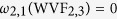 for 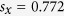. Note that the events 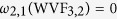 at 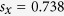 and 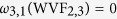 at 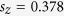 do not reverse the rotational direction of the respective wavy flows (See, Figure 3).

Figure 4 shows the variation in the radial velocities of the wavy flows in the presence of an applied transverse 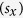 or axial 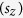 magnetic field. In order to characterize the flow structures, we examine radial flow field amplitudes  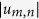  at mid-gap and display the contributions from the dominant, axisymmetric 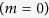 mode, as well as those from the dominant non-axisymmetric (*m* ≠ 0) modes embedded in the underlying flow structure. For the parameter setting in Fig. 4, the dominant modes are 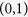, 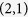 and 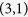 for either WVF_2,3_ and WVF_3,2_ flows. We see that a purely axial field *does not change* the structure of the flow pattern in the real space nor the mode structure in the Fourier plane (*m*, *n*)^17^,^18^. In contrast, when the magnetic field has a finite transverse component *s*_*x*_≠ 0, the structures are *changed* due to excitation of higher-order modes 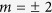, as described in detail in ref. 17.

For increasing axial field strength 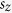, all mode amplitudes for WVF_2,3_ and WVF_3,2_ decrease monotonically, as shown in Fig. 4(a). However, as the transverse component of the magnetic field is strengthened, all the mode amplitudes increase monotonically, as shown in Fig. 4(b). In fact, similar behaviors occur for the unstable TVFs. Examining the corresponding frequencies of the complex mode amplitudes [Fig. 4(c,d)], we see that 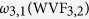
’s approaching zero at 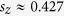 or 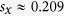 exhibits a similar behavior with respect to axial or transverse magnetic field: it is the critical point at which the rotational direction of the wavy flow pattern WVF_3,2_ reverses. For sufficiently large value of 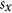, 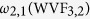 crosses zero and becomes negative, enforcing the entire flow pattern in the retrograde direction and resulting in a large negative value of the angular velocity Ω [cf., Fig. 3(d)]. We also find that the angular velocity Ω of WVF_2,3_ is determined by the two frequencies 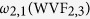 and 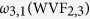. The first reversal in the flow pattern direction is associated with the vanishing of 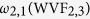 at 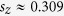, and the second reversal occurs when 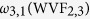 approaches zero for the second time at 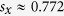. The first time that 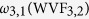 becomes zero at 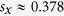 only leads to an enhancement of the retrograde behavior with no effect on the flow pattern direction. The frequency 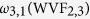 reaches its minimum at 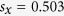, which is different from the value of 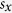 for which the angular velocity is minimized [cf., Fig. 3]. The reason is that both frequencies 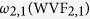 and 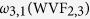 are negative but the magnitude of 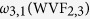 is larger. A general observation is that increasing 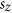 reduces the flow complexity but an increase in 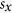 plays the opposite role, i.e., making the flow more complex.

### Flow patterns in an axial magnetic field

**Figure 5:**
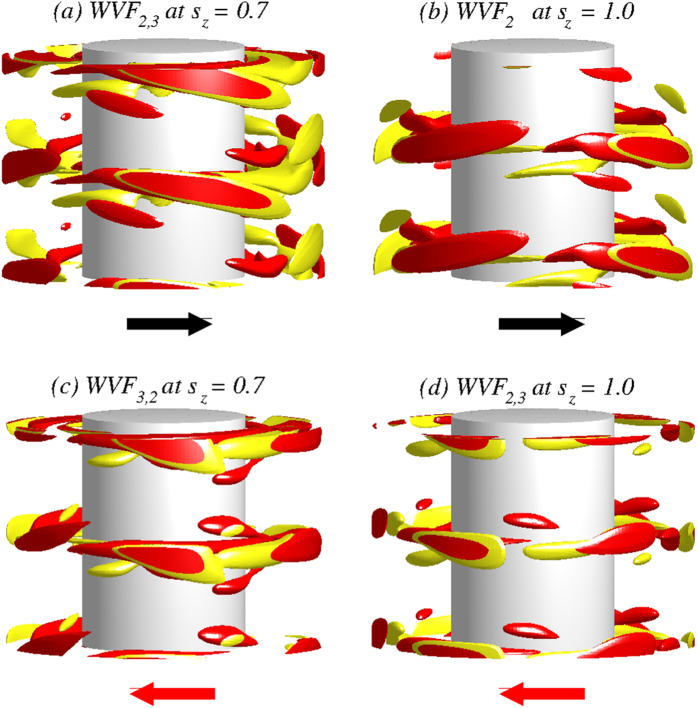
Structural properties of flows in the presence of an axial magnetic field. Isosurfaces of 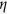 for WVF_2,3_, WVF_3,2_ and WVF_2_ for 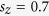 and 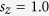. Vorticity isosurfaces are for 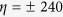, and two periods of motion are plotted in the axial direction. The arrows below the snapshots illustrate the rotational direction, which appears laterally on the cylinders. For these values of 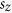 the flow pattern of WVF_2,3_ is pro-grade but that of WVF_3,2_ is retrograde. See also movie files movie3.avi and movie4.avi in SMs.

Figure 5 shows the isosurfaces of the azimuthal vorticity *η* over two axial wavelengths for wavy flows at two values of the axial magnetic field strength: 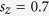 and 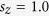 [See movie files movie3.avi and movi4.avi in SM.]. The spatiotemporal structures are qualitatively the same as for the case without any magnetic field (Fig. 1). As 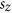 is increased, modulations in all flow structures become weaker. The 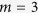 contribution to the flow is either reduced (Fig. 4) or vanishes completely, as is visible from the pattern of WVF_2_ for 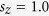. In general, an axial magnetic field weakens the waviness of the underlying flows.

**Figure 6:**
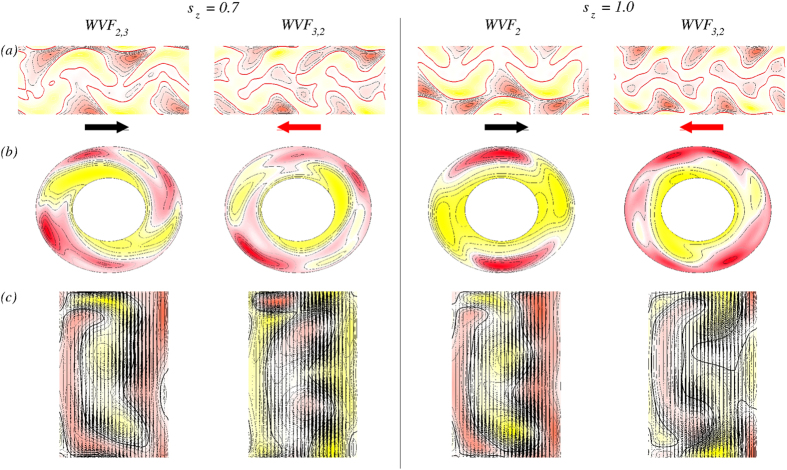
Flows in the presence of an axial magnetic field (cf.Fig. 5). Contours of flows for 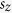 values as indicated. (***a***) Contour of the radial velocity 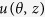 on an unrolled cylindrical surface in the annulus at mid-gap. The arrows below illustrate the rotational direction. (***b***) Contour of the velocity component 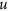 in the 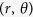 plane at mid-height. (***c***) Vector plots 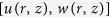 of the radial and axial velocity components in a constant *θ* plane, with color-coded azimuthal vorticity *η* from red (minimum) to yellow (maximum). See also movie files movie3.avi and movie4.avi in SMs.

Figure 6 shows the contour plots for the same wavy flows and values of 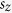 as in Fig. 5. As shown in Fig. 6(a), the behavior of the radial velocity 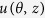 on an unrolled cylindrical surface at mid-gap demonstrates a mixture of the azimuthal modes: 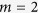 and 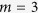 for WVF_2,3_ and WVF_3,2_. The corresponding plot for WVF_2_ contains the 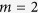 mode as the only non-axisymmetric component. The symmetries are apparent in the contour plots of the azimuthal vorticity in the 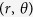 plane, as shown in Fig. 6(b). The clear two-fold symmetry can be seen for WVF_2_. The predominance of the 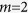 mode can also be seen from the behavior of WVF_2,3_ [Fig. 6(a)], but such a dominance is not apparent in WVF_3,2_ [Fig. 6(b)] because it contains contributions from both 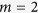 and 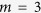 modes [cf., Fig. 4]. For 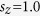, WVF_3,2_ is dominated by the 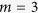 mode. From the vector plots of the radial and axial velocity 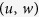 on an 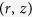 plane with the color-coded azimuthal vorticity, we see that the center of the vortices move outward for WVF_3,2_ with respect to WVF_2_ and WVF_2,3_.

### Flow patterns in a transverse magnetic field

**Figure 7:**
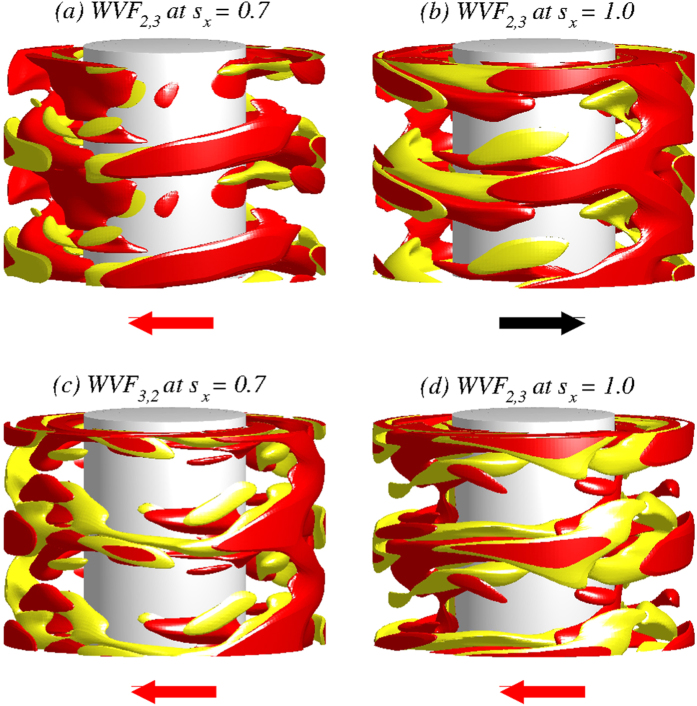
Flow structures in a transverse magnetic field. Isosurfaces of *η* for WVF_2,3_ and WVF_3,2_ for transverse magnetic field strength 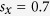 and 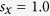. Vorticity isosurfaces are 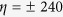 and two periods are plotted in the axial direction. The arrows below the snapshots illustrate the rotational direction (appearing laterally on the cylinders). For 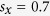, the WVF_2,3_ flow is pro-grade but for 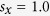, it is retrograded. The WVF_3,2_ flow is retrograde for both values of 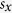 (cf. Fig. 5). See also movie files movie5.avi, movie6.avi and movie7.avi in SMs.

**Figure 8:**
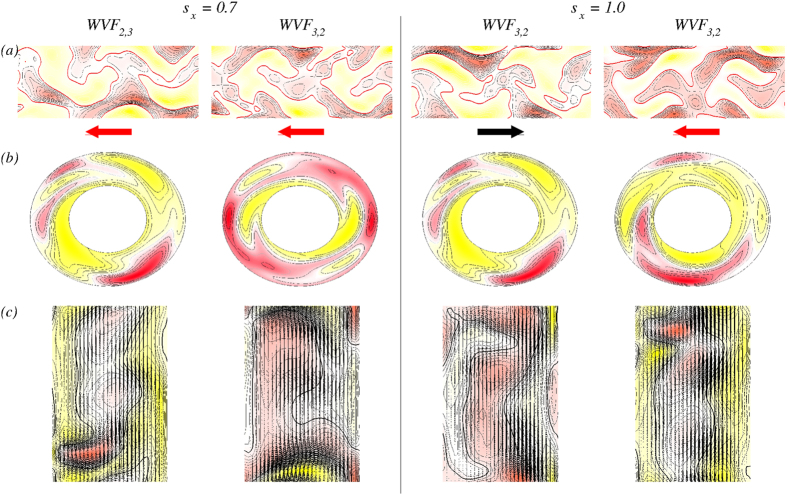
Flow structures in a transverse magnetic field (cf.Fig. 7). (***a***) Contours of the radial velocity 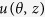 on an unrolled cylindrical surface in the annulus at mid-gap. The arrows below illustrate the rotational direction. (***b***) Contours of the velocity component *u* in the 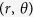 plane at mid-height. (***c***) Vector plots 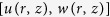 of the radial and axial velocity component in a constant *θ* = plane, with color coded azimuthal vorticity *η* from red (minimum) to yellow (maximum). See also movie files movie5.avi, movie6.avi and movie7.avi in SMs.

Figure 7 shows the isosurfaces of the azimuthal vorticity *η* over two axial wavelengths for wavy flows for transverse magnetic field strength 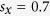 and 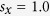. [See movie files movie5.avi, movie6.avi and movie7.avi in SM.] Compared with the structures under an axial magnetic field, [Fig. 5], we observe more complex wavy flows. The isosurfaces of 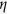 are much more intertwined in a transverse magnetic field compared to the ones in an axial magnetic field [Fig. 5]. The increased complexity can be better seen in the contour plots in Fig. 8 for WVF_2,3_ and WVF_3,2_. The plots of the radial velocity 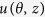 on an unrolled cylindrical surface at mid-gap [Fig. 8(a)] illustrate the more complex flow structures in the transverse magnetic field due to the azimuthal modes 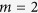 and 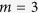 in the WVF_2,3_ and WVF_3,2_ flow. For 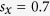, both WVF_2,3_ and WVF_3,2_ contain a strong contribution from the 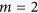 mode, which is visible in the azimuthal vorticity plot in the 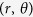 plane [Fig. 8(b)]. In fact, WVF_3,2_ for 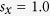 does not exhibit any predominant symmetry due to similar amplitudes of the 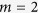 and 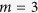 modes [Fig. 4(b)]. In general, the increase in the complexity for larger field strength 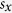 can be attributed to the enhanced mode amplitudes (cf., Fig. 4) resulting from the intrinsic stimulation of higher 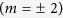 modes under a transverse magnetic field^17^.

### Behavior of the angular momentum and torque

**Figure 9:**
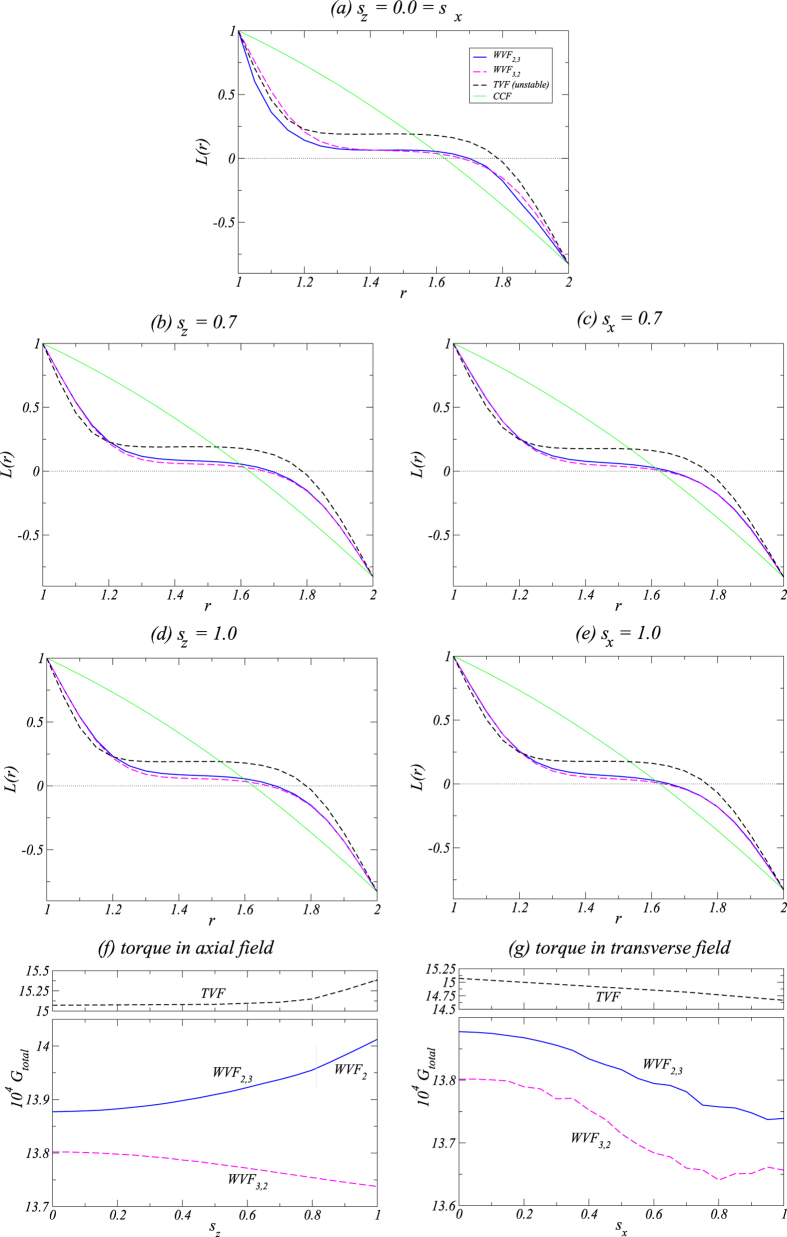
Angular momentum and torque. (***a*****–*****e***) Angular momentum 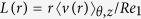 scaled with the inner Reynolds number versus the radius *r* for the solutions: WVF_2,3_ (WVF_2_ for 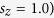, WVF_3,2_, unstable TVF, and CCF for 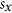 and 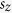 values as indicated. (***f***,***g***) Variation with 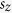 and 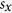 of the dimensionless torque 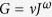 (see text for details) for WVF_2,3_ (WVF_2_) and WVF_3,2_.

To better characterize the flow pattern reversal phenomenon, we examine the behaviors of the angular momentum and torque for a variety of flow structures. Figure 9(a–e) show the mean (axially and azimuthally averaged) angular momentum 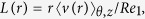 scaled with the inner Reynolds number, versus the radius *r* for three different combinations of the magnetic field strength 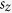 and 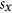, respectively. The black short dashed curve shows the angular momentum for the unstable TVF and the green thin solid line is for the unstable equilibrium circular Couette flow (CCF), respectively. Particularly, Fig. 9(a) is for the case of zero magnetic field 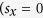 and 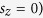. The TVF and wavy flows show the typical behavior that the angular momentum is transported outwards from the inner cylinder. All curves have similar shape with increased slope (gradient) of 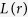 near the boundaries and reduced gradient in the interior. For any combination of the field strength 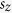 and 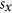, the unstable TVF has the steepest gradient of 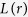 at the outer boundary, due to the stronger torque on the outer cylinder than the wavy flows and CCF. For sufficiently large values of the magnetic field strength, e.g., 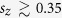 or 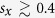, the unstable TVF has the steepest slope at the inner boundary layer, as shown in Fig. 9(a–e).

In the absence of any magnetic field [Fig. 9(a)], the gradient of 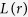 near the inner cylinder is the largest for WVF_2,3_, the smallest for WVF_3,2_, and intermediate for the unstable TVF. In this case, WVF_2,3_ (WVF_3,2_) has the largest (smallest) torque on the inner cylinder. For WVF_3,2_ there is a plateau in the gradient near the central region, but the unstable TVF exhibits the plateau at larger values of 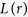. In the presence of an axial or a transverse magnetic field, the gradients of 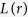 for both types of wavy flows are quite similar and differ only slightly in the fluid interior. In general the plateaus for WVF_2,3_ are at slightly higher values 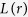 compared with those for WVF_3,2_, with larger differences in an axial than in a transverse magnetic field. Near the boundaries the difference diminishes. In general, a magnetic field decreases the gradient of 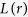 near the inner cylinder, consequently reducing the torque on the boundary layers.

The behaviors of dimensionless torque 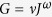 with field strength 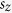 and 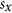 are shown in Fig. 9(g). In calculating the torque we used the fact that for a flow between infinite cylinders the transverse current of the azimuthal motion, 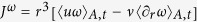 (with 
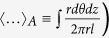
, is a conserved quantity^29^. Thus the dimensionless torque is the same at the inner and the outer cylinders. As the axial magnetic field strength 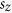 is increased, the torque *G* decreases monotonically for WVF_3,2_ but it increases monotonically for WVF_2,3_ and unstable TVF. When a transverse magnetic field is applied, *G* decreases with 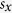 for all flow types examined. We thus see that, while either an axial or a transverse magnetic field can stabilize the basic flow states, their effects on the dynamical behaviors of the flows can be quite different.

## Conclusions

The phenomenon of flow pattern reversal is interesting as it is relevant to intriguing natural phenomena such as geomagnetic reversal. We study computationally the reversal of ferrofluidic wavy flows in the classic counter-rotating Taylor-Couette system. In absence of any magnetic field all wavy flows are pro-grade in the sense that they rotate in the same direction as the inner cylinder. However, an axial or a transverse magnetic field can slow down the flow, leading to standing waves and subsequently to reversal into a retrograde flow state. For an axial magnetic field, there can be at most one reversal. However, for a transverse magnetic field, a second reversal can occur at which the flow pattern becomes pro-grade again. All these can occur when a single parameter, the magnetic field strength, is increased. We elucidate the structural properties of the flow by examining the variations in the significant mode amplitudes and frequencies with the magnetic field. A general finding is that a transverse magnetic field can be more effective in generating flow pattern reversal, due to the stimulation of higher modes^17^ (cf., Figs 4 and 7).

**Figure 10:**
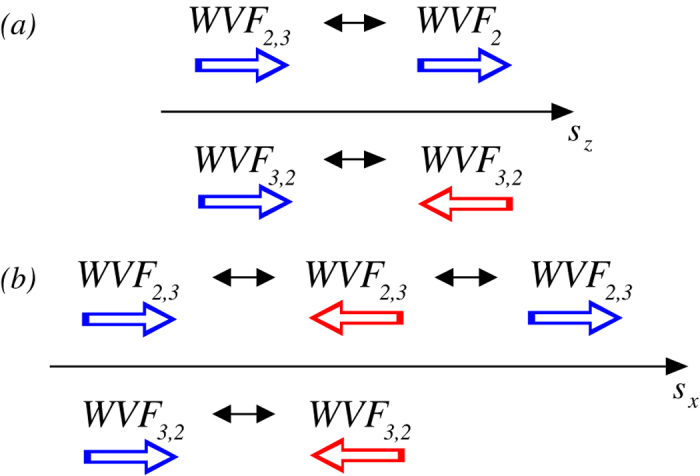
Summary: schematic illustration of flow reversal with magnetic field strength: (***a***) axial field and (***b***) transverse field. Thick arrows below the terms indicate the rotating directions of the corresponding flow patterns, which appear lateral on the cylinder. Note that the sequences hold for increase and decrease of magnetic field strength.

Figure 10 summarizes our findings in terms of the sequences of wavy flow patterns with either increasing axial magnetic field strength 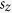 or increasing transverse magnetic field strength 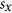, where large arrows indicate the rotating directions of the corresponding flow pattern, which appear lateral on the cylinder, and the sequences are the same for small 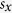 and 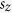 values. We see that WVF_3,2_ has a single reversal in its direction of motion with 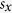 or 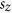. WVF_2,3_ does not exhibit any flow pattern reversal under an axial magnetic field but it changes the rotating direction *twice* with continuous increase in the strength 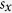 of a transverse magnetic field.

It may be interesting to investigate the effects of combined axial and transversal magnetic field. It has been known that interactions among the modes can increase the flow complexity^17^. It may also be useful to study more realistic system sizes because previous experimental^20^ and computational^19^ studies revealed that an applied magnetic field can change the number of vortices, or the wavenumber, in the bulk. It would be insightful to study whether this can occur for wavy flows, especially in terms of the effects on the azimuthal component. Moreover the effects of magnetic fields on other wavy flows with different topology as helical wavy spiral states^30^ may be interesting, particularly for realisic axial boundary conditions.

We hope that our computational results will stimulate experimental works on ferrofluidic wavy flows. Since the setting of our computation and the choices of the simulation parameters are experimentally motivated, it may be feasible to realize flow pattern reversal in experiments. For example, our ferrofluid APG933 and the typical magnetic field of 100 [*kA*/*m*] (about 0.968 in terms of 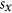 or 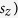 are realizable in laboratories. Exploiting other ferrofluids such as those based on Cobol can reduce the required magnetic field strength^20^. Control of flow pattern reversal through variations of the external magnetic field appears promising.

## Methods

### Ferrohydrodynamical equation of motion

Consider a TCS consisting of two concentric, independently rotating cylinders with an incompressible, isothermal, homogeneous, mono-dispersed ferrofluid of kinematic viscosity *v* and density *ρ* within the annular gap. The inner and outer cylinders of radii 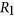 and 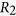 rotate at the angular speeds 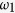 and 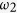, respectively. The boundary conditions at the cylinder surfaces are of the non-slip type but axially periodic boundary conditions of period (length) Γ are used. The system can be described in the cylindrical coordinate system 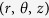 with the velocity field 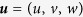 and the corresponding vorticity 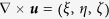. We set the radius ratio of the cylinders and the parameter Γ to typical values used in experiments, e.g., 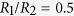 and 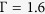, where the latter corresponds to an axial wavenumber 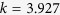. A homogeneous magnetic field 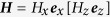 is applied, where 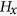 and 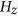 are the field strengths in the transverse 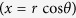 and axial directions (*z*), respectively. To keep the setting as simple as possible for uncovering new phenomena, we assume that the magnetic field is either transverse or axial, i.e., either 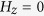 or 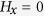, as the presence of both transverse and axial components in the field can give rise to unnecessary complications^17^. The gap width 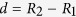 is chosen as the length scale and the diffusion time 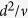 serves as the time scale. The pressure is normalized by 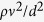, and the magnetic field **H** and magnetization *M* can be normalized by the quantity 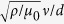, where 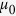 is the permeability of free space. These considerations lead to the following non-dimensionalized hydrodynamical equations^19^,^31^:


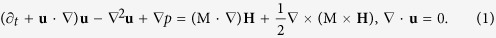


The boundary conditions on the cylindrical surfaces are 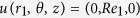 and 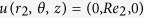, where the inner and outer Reynolds numbers are 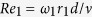 and 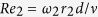, respectively, 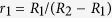 and 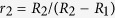  are the non-dimensionalized inner and outer cylinder radii, respectively. To be concrete, we consider counter-rotating cylinders and fix the Reynolds numbers at 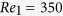 and 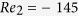. The rotation ratio 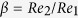 of the cylinders is about −0.4143.

We need to solve Eq. (1) together with an equation that describes the magnetization of the ferrofluid. Using the equilibrium magnetization of an unperturbed state where homogeneously magnetized ferrofluid is at rest and the mean magnetic moment is orientated in the direction of the magnetic field, we have 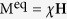. The magnetic susceptibility 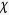 of the ferrofluid can be approximated with the Langevin’s formula^32^, where we set the initial value of 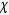 to be 0.9 and use a linear magnetization law. The ferrofluid studied corresponds to APG933^33^. We consider the near equilibrium approximations of Niklas^34^,^35^ with small 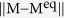 and small magnetic relaxation time *τ*: 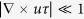. Using these approximations, one can obtain^19^ the following magnetization equation:


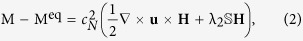


where


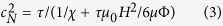


is the Niklas coefficient^34^, *μ* is the dynamic viscosity, 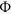 is the volume fraction of the magnetic material, 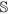 is the symmetric component of the velocity gradient tensor^19^,^31^, and 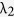 is the material-dependent transport coefficient^31^, which we choose to be 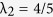
17,31,36. Using Eq. (2), we can eliminate the magnetization from Eq. (1) to obtain the following ferrohydrodynamical equations of motion^19^,^31^:


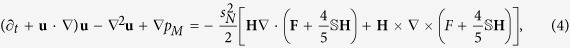


where 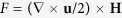, 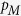 is the dynamic pressure incorporating all magnetic terms that can be expressed as gradients, and 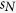 is the Niklas parameter [Eq. (6)]. To the leading order, the internal magnetic field in the ferrofluid can be approximated as the externally imposed field^37^, which is reasonable for obtaining dynamical solutions of the magnetically driven fluid motion. Equation (4) can then be simplified as


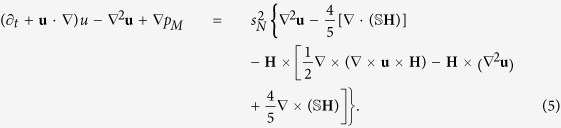


This way, the effect of the magnetic field and the magnetic properties of the ferrofluid on the velocity field can be characterized by a single parameter, the magnetic field or the Niklas parameter^34^, 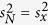 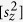, with


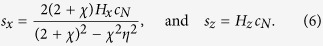


### Numerical scheme for ferrohydrodynamical equation

The ferrohydrodynamical equations of motion Eq. (4) can be solved^17^,^19^,^37^ by combining a second-order finite-difference scheme in 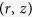 with Fourier spectral decomposition in *θ* and (explicit) time splitting. The variables can be expressed as


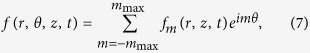


where *f* denotes one of  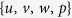. For the parameter regimes considered, the choice 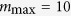 provides adequate accuracy. We use uniform grids with spacing 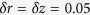 and time-steps 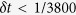. For diagnostic purposes, we also evaluate the complex mode amplitudes 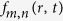 obtained from the Fourier decomposition in the axial direction 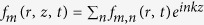, where 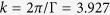.

## Additional Information

**How to cite this article**: Altmeyer, S. *et al.* Magnetic field induced flow pattern reversal in a ferrofluidic Taylor-Couette system. *Sci. Rep.* **5**, 18589; doi: 10.1038/srep18589 (2015).

## Supplementary Material

Supplementary Information

Supplementary Movie 1

Supplementary Movie 2

Supplementary Movie 3

Supplementary Movie 4

Supplementary Movie 5

Supplementary Movie 6

Supplementary Movie 7
