# Supplementary material for: Magnetic field induced flow pattern reversal in a ferrofluidic Taylor-Couette system
Source: Sci Rep. 2015 Dec 21;5:18589. doi: 10.1038/srep18589 (PMC4685649; doi:10.1038/srep18589)
Supplement: Supplementary Information [file srep18589-s1.pdf]

Supplementary Material to "Magnetic field induced flow pattern reversal in a ferrofluidic Taylor-Couette system"

Sebastian Altmeyer,[1] Younghae Do,[2] and Ying-Cheng Lai[3]

[1]*Institute of Science and Technology Austria (IST Austria), 3400 Klosterneuburg, Austria*
[2]*Department of Mathematics, KNU-Center for Nonlinear Dynamics, Kyungpook National University, Daegu, 702-701, South Korea*
[3]*School of Electrical, Computer and Energy Engineering, Arizona State University, Tempe, Arizona, 85287, USA*

(Dated: October 31, 2015)

Legends for videos in SM

- Movie 1:
  Movie 1 demonstrates *pro-grade* $\mathrm{WVF}_{2,3}$ in absence of a magnetic field ($s_x = 0.0, s_z = 0.0$). *Top left panel:* isosurfaces of the azimuthal voticity $\eta = \partial_z u_r - \partial_r u_\theta = \pm 240$ (red: $\eta = 240$, yellow: $\eta = -240$). *Top right panel:* radial velocity $u(\theta, z)$ on an unrolled cylindrical surface in the annulus at mid-gap. *Bottom left panel:* azimuthal vorticity η in the (r, θ) plane at mid-height.

- Movie 2:
  Movie 2 demonstrates *pro-grade* $\mathrm{WVF}_{3,2}$ in absence of a magnetic field ($s_x = 0.0, s_z = 0.0$). *Top left panel:* isosurfaces of the azimuthal voticity $\eta = \partial_z u_r - \partial_r u_\theta = \pm 240$ (red: $\eta = 240$, yellow: $\eta = -240$). *Top right panel:* radial velocity $u(\theta, z)$ on an unrolled cylindrical surface in the annulus at mid-gap. *Bottom left panel:* azimuthal vorticity η in the (r, θ) plane at mid-height.

- Movie 3:
  Movie 3 demonstrates *retrograde* WVF_2 at $s_x = 0.0$ and $s_z = 1.0$. *Top left panel:* isosurfaces of the azimuthal voticity $\eta = \partial_z u_r - \partial_r u_\theta = \pm 240$ (red: $\eta = 240$, yellow: $\eta = -240$). *Top right panel:* radial velocity $u(\theta, z)$ on an unrolled cylindrical surface in the annulus at mid-gap. *Bottom left panel:* azimuthal vorticity η in the (r, θ) plane at mid-height.

- Movie 4:
  Movie 4 demonstrates *retrograde* $\mathrm{WVF}_{3,2}$ at $s_x = 0.0$ and $s_z = 1.0$. *Top left panel:* isosurfaces of the azimuthal voticity $\eta = \partial_z u_r - \partial_r u_\theta = \pm 240$ (red: $\eta = 240$, yellow: $\eta = -240$). *Top right panel:* radial velocity $u(\theta, z)$ on an unrolled cylindrical surface in the annulus at mid-gap. *Bottom left panel:* azimuthal vorticity η in the (r, θ) plane at mid-height.

- Movie 5:
  Movie 5 demonstrates *retrograde* $\mathrm{WVF}_{2,3}$ at $s_x = 0.7$ and $s_z = 0.0$. *Top left panel:* isosurfaces of the azimuthal voticity $\eta = \partial_z u_r - \partial_r u_\theta = \pm 240$ (red: $\eta = 240$, yellow: $\eta = -240$). *Top right panel:* radial velocity $u(\theta, z)$ on an unrolled cylindrical surface in the annulus at mid-gap. *Bottom left panel:* azimuthal vorticity η in the (r, θ) plane at mid-height.

- Movie 6:
  Movie 6 demonstrates *pro-grade* $\mathrm{WVF}_{2,3}$ at $s_x = 1.0$ and $s_z = 0.0$. *Top left panel:* isosurfaces of the azimuthal voticity $\eta = \partial_z u_r - \partial_r u_\theta = \pm 240$ (red: $\eta = 240$, yellow: $\eta = -240$). *Top right panel:* radial velocity $u(\theta, z)$ on an unrolled cylindrical surface in the annulus at mid-gap. *Bottom left panel:* azimuthal vorticity η in the (r, θ) plane at mid-height.

- Movie 7:
  Movie 7 demonstrates *retrograde* $\mathrm{WVF}_{3,2}$ at $s_x = 1.0$ and $s_z = 0.0$. *Top left panel:* isosurfaces of the azimuthal voticity $\eta = \partial_z u_r - \partial_r u_\theta = \pm 240$ (red: $\eta = 240$, yellow: $\eta = -240$). *Top right panel:* radial velocity $u(\theta, z)$ on an unrolled cylindrical surface in the annulus at mid-gap. *Bottom left panel:* azimuthal vorticity η in the (r, θ) plane at mid-height.
